# Surgical Anatomy

**Published:** 1850-06

**Authors:** 


ART. VII.—*Surgical Anatomy. Part* 2. By JOSEPH MACLISE, Surgeon. With colored plates. Philadelphia: Lea & Blanchard. 1850.

We have already announced the character and terms of this publication, and we renew a reference to the terms. The work will be published in four parts, containing twelve to sixteen colored plates each, the whole forming one large imperial quarto volume, with about one hundred and fifty double-columned pages.

This is by far the ablest work on Surgical Anatomy that has come under our observation. The London Lancet says, of it: "The practical knowledge of the surgeon, the patience and skill of the dissector, in combination with the genius of the artist, as here displayed, have never before been, and perhaps never will be again associated to a similar extent in the same individual." We cordially join in the whole of this commendation. We knew of no other work that would justify a student, in any degree, for neglect of actual dissection. A careful study of these plates, and of the commentaries on them, would almost make an anatomist of a diligent student. And to one who has studied anatomy, by dissection, this work is invaluable, as a perpetual remembrancer, in matters of knowledge that may slip from the memory. The practitioner can scarcely consider himself equipped for the duties of his profession, without such a work as this, and this has no rival, in his library. In those sudden emergencies that so often arise, and which require the instantaneous command of minute anatomical knowledge, a work of this kind keeps the details of the dissecting room, perpetually fresh in the memory. We appeal to our readers, whether any one can justifiably undertake the practice of medicine, who is not prepared to give all needful assistance, in all matters demanding immediate relief.

We repeat that no medical library, however large, can be complete without Maclise's Surgical Anatomy. The American edition is well entitled to the confideuce of the profession, and should command, among them, an extensive sale. The investment of the amount of the cost of this work, will prove to be a very profitable one, and if practitioners would qualify themselves, thoroughly, with such important knowledge as is contained in works of this kind, there would be fewer of them sighing for employment. The medical profession should spring towards such an opportunity as is presented in this republication, to encourage frequent repetitions of American enterprise of this kind.

